# Pleth Variability Index predicts fluid responsiveness in critically ill patients

**DOI:** 10.1186/cc9496

**Published:** 2011-03-11

**Authors:** H Nanadoumgar, TL Loupec, DF Frasca, FP Petitpas, LL Laksiri, DB Baudouin, OM Mimoz

**Affiliations:** 1 CHU Poitiers, France

## Introduction

In patients with acute circulatory failure related to sepsis or hypovolemia, volume expansion is used as first-line therapy in an attempt to improve cardiac output. Dynamic indices based on cardiopulmonary interactions and variation in left ventricular stroke volume like respiratory variations in arterial pulse pressure (ΔPP) are able to predict response to fluid loading in mechanically ventilated patients. The Pleth Variability Index (PVI) (Masimo^® ^Corp., Irvine, CA, USA) is a new non-invasive technique based on perfusion index (PI) variations during the respiratory cycle in mechanically ventilated patients. The objective of the study is to investigate whether PVI, a non-invasive and continuous tool, can predict fluid responsiveness in mechanically ventilated patients with circulatory insufficiency.

## Methods

A prospective study in a surgical ICU of a university hospital. Forty mechanically ventilated patients with circulatory insufficiency were included in whom volume expansion was planned by the attending physician. Exclusion criteria included spontaneous respiratory activity; cardiac arrhythmia; known intracardiac shunt; severe hypoxemia (PaO_2_/FIO_2 _< 100 mmHg); contraindication for passive leg raising (PLR); altered left ventricular ejection fraction; hemodynamic instability during the procedure. We performed fluid challenge with 500 ml of 130/0.4 hydroxyethylstarch if ΔPP ≥13% or with PLR otherwise. PVI, ΔPP and cardiac output (CO) estimated by echocardiography were recorded before and after fluid challenge. Fluid responsiveness was defined as an increase in CO ≥15%.

## Results

Twenty-one patients were responders and 19 were non-responders. Median (interquartile range) PVI (26% (20 to 34%) vs. 10% (9 to 14%)) and ΔPP (20% (15 to 29%) vs. 5% (3 to 7%)) values at baseline were significantly higher in responders than in nonresponders. A PVI threshold value of 17% allowed discrimination between responders and nonresponders with a sensitivity of 95% (95% CI = 74 to 100%) and a specificity of 91% (95% CI = 70 to 99%). PVI at baseline correlated (*r *= 0.72; *P *< 0.0001) with percentage changes in CO (ΔCO) induced by fluid challenge, suggesting the higher PVI at baseline, the higher ΔCO after volume expansion.

## Conclusions

PVI can predict fluid responsiveness non-invasively in ICU patients under mechanical ventilation.

